# A Computer-Aided Pipeline for Automatic Lung Cancer Classification on Computed Tomography Scans

**DOI:** 10.1155/2018/9409267

**Published:** 2018-11-01

**Authors:** Emre Dandıl

**Affiliations:** Department of Computer Engineering, Faculty of Engineering, Bilecik Seyh Edebali University, Gulumbe Campus, 11210 Bilecik, Turkey

## Abstract

Lung cancer is one of the most common cancer types. For the survival of the patient, early detection of lung cancer with the best treatment method is crucial. In this study, we propose a novel computer-aided pipeline on computed tomography (CT) scans for early diagnosis of lung cancer thanks to the classification of benign and malignant nodules. The proposed pipeline is composed of four stages. In preprocessing steps, CT images are enhanced, and lung volumes are extracted from the image with the help of a novel method called lung volume extraction method (LUVEM). The significance of the proposed pipeline is using LUVEM for extracting lung region. In nodule detection stage, candidate nodules are determined according to the circular Hough transform- (CHT-) based method. Then, lung nodules are segmented with self-organizing maps (SOM). In feature computation stage, intensity, shape, texture, energy, and combined features are used for feature extraction, and principal component analysis (PCA) is used for feature reduction step. In the final stage, probabilistic neural network (PNN) classifies benign and malign nodules. According to the experiments performed on our dataset, the proposed pipeline system can classify benign and malign nodules with 95.91% accuracy, 97.42% sensitivity, and 94.24% specificity. Even in cases of small-sized nodules (3–10 mm), the proposed system can determine the nodule type with 94.68% accuracy.

## 1. Introduction

Nowadays, lung cancer is one of the ranking first causes of mortality worldwide among men and women [1, 2]. Although there are a lot of treatment options like surgery, radiotherapy, and chemotherapy, five year survival rate for patients is quite low [3]. However, survival rate may go up to 54% in case lung cancer is identified in an early stage [4]. Therefore, early detection of lung cancer is vital to decrease lung cancer mortality.

Medical imaging techniques have been important technology in screening of lung cancer recently. CT scan becomes a standard modality for detecting and assessing lung cancer [5]. Most of the lung nodules are usually benign. However, some nodules such as calcified, swollen, and hard can also be determined as benign. Similarly, a hard nodule generally is cancerous (malignant), but it may be considered as benign case in some cases [6]. Furthermore, medical CT images are needed to be diagnosed by radiologists.

Computer-aided detection (CAD) systems have been an important field in medical image processing. CAD systems also based on machine learning methods designed to diagnosis of cancer have become common in recent years. Radiologists and physicians may use findings of CAD systems as the second opinion before making their own final decisions. Therefore, CAD systems play an important role in CT scans to help radiologists for detection of lung cancer efficiently.

## 2. Related Work

Computer-aided detection (CAD) systems have been active research field for the pulmonary nodule detection and malign/benign nodule classification. Until now, many CAD systems have been proposed. For example, Ozekes and Camurcu proposed a method for pulmonary nodule detection method using template matching [7]. Schilham et al. presented a CAD system which consists of image preprocessing, candidate nodule detection, feature extraction, and classification for nodule detection in chest radiographs [8]. Dehmeshki et al. detected lung nodules using shape-based genetic algorithm template matching [9]. Suarez-Cuenca et al. also designed a system which discriminates the nodules and non-nodule cases using iris filter in CT images [10]. Murphy et al. automatically performed lung nodule detection using k-nearest neighbours classifier [11]. Giger et al. realized CAD system to detect lung nodules on CT images using geometric features [12]. In addition, Hasegawa et al. proposed image processing methods for identification of lung nodules using CT scans [13]. In another study, Kanazawa et al. used a CAD system to identify pulmonary nodules with fuzzy features [14]. In 2005, Suzuki et al. proposed a method using ANN for classification of malignant and benign nodules on CT images [15]. Sun et al. compared support vector machines (SVM) with the some classification methods for detection of lung cancer on CT images [16]. Kuruvilla and Gunavathi proposed a system using ANNs for classification of lung cancer [17].

In a recent study on lung nodule detection, Javaid et al. proposed a computer aided nodule detection method for the segmentation and detection of challenging in different type nodules [18]. ur Rehman et al. presented a systematic analysis of nodules detection techniques with the current trends and future challenges [19]. Wang et al. proposed a pulmonary nodule CAD based on semisupervised extreme learning machine [20]. Xie et al. proposed an automated pulmonary nodule detection system with 2D convolutional neural network (CNN) on LUNA16 dataset [21].

In this study, we have proposed fully automated computer-aided pipeline for the detection of pulmonary nodules and classification of benign/malign nodules in early stage. The contributions of this paper are (1) to review the systematic literature review; (2) to present the state of the art detection of pulmonary nodules and classification of lung cancer; (3) to propose the novel preprocessing method (LUVEM) for the lung volume extraction; (4) to suggest a novel candidate lung nodule detection method using CHT; (5) to design a holistic pipeline for the detection of pulmonary nodules as well as lung cancer; (6) the detailed comparison of feature extraction methods for lung nodule detection; and (7) to perform the detailed performance evaluation, high true detection rate, and low false detection rate for nodule detection and classification.

## 3. Architecture of the Computer-Aided Pipeline

**Figure 1:**
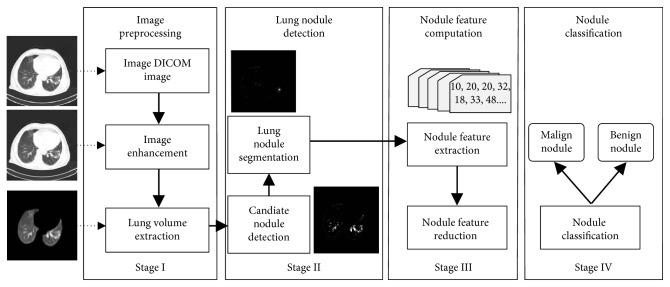
Work flow of the designed pipeline to detect lung cancer. The system consists of four stages: Stage I—enhancement of lung CT image and a novel lung volume segmentation method (LUVEM), Stage II—candidate nodule detection using CHT and segmentation of lung nodules using SOM, Stage III—computing of lung nodule features and reduction features using PCA, and Stage IV—classification of malign and benign lung nodule using PNN.

Designed pipeline consists of four main stages such as image preprocessing (Stage I), lung nodule detection (Stage II), nodule feature computation (Stage III), and nodule classification (Stage IV). The work flow of the pipeline is presented in Figure 1.

### 3.1. Lung Image Preprocessing

In the first step of the image preprocessing stage, reading of CT images is performed. The CT scans obtained for the work are stored as DICOM (Digital Imaging and Communications in Medicine) files [22].

The goal of image enhancement step is to prevent misleading results that may occur in subsequent processes. Thus, we firstly implemented the median filter to remove unnecessary noises and enhance the images. Moreover, the sharpening of nodule contours is an important step for the detection of nodules. Laplacian filter was used in our study. So, nodules on lung region were able to be detected more accurately. Furthermore, histogram equalization was also used in enhancement step in order to minimize contrast differences which occur due to scanning errors and to remove unnecessary grains.

In lung volume extraction step of image preprocessing stage, extracting of the lung region from CT image is performed. There are many methods for extracting lung volume from a lung CT scan [9, 10, 23–25]. However, these methods are complex, and they require more processing overhead. In some cases, these methods may lead to losses of information about lung regions or cause noise. The purpose of this step is to extract the lung region completely from the full lung CT image. Therefore, a simple but effective and novel method has been proposed in this study for lung volume extraction named as lung volume extraction method (LUVEM). The pseudocode of LUVEM is shown in Algorithm 1.

**Figure 2:**
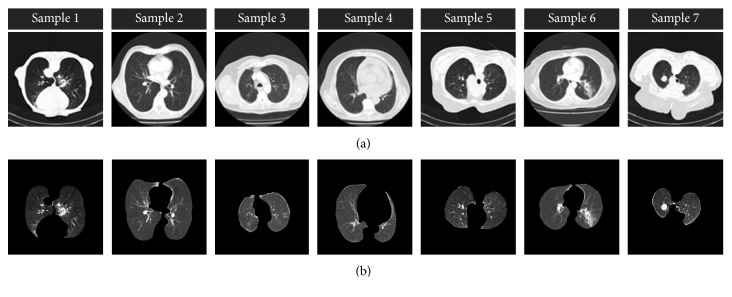
Extraction examples of lung regions: (a) preprocessed images and (b) lung volume extraction using LUVEM.

In LUVEM method, lung lobes are extracted from CT images with the help of morphological operations. LUVEM removes unrelated segments on the sides and edges of the preprocessed image and obtain the lung region successful. In the algorithm, input image is firstly converted to double-formatted image. Afterwards, 1 or 0 values are assigned to each pixel of double-formatted image according to low and high threshold values. The low and high threshold values are determined 0.25 and 0.65 in this algorithm, respectively. The method removes the bright areas on the edges of the lung CT image since their average values change between low and high values. After this process, the image is converted to binary format and performed morphological operations which are eroding, dilating, and filling, respectively. Finally, the image is again converted to gray-scale format. The segmentation examples of LUVEM can be seen in Figure 2. It is clearly seen that LUVEM can successfully extract the lung volume. In addition, quantitative evaluation of LUVEM will be reported below.

### 3.2. Lung Nodule Detection

**Figure 3:**
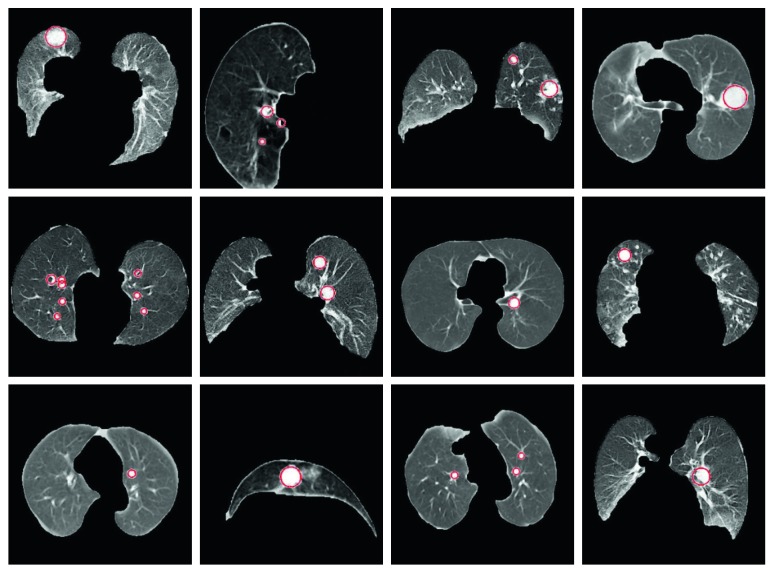
Determination of candidate lung nodule using CHT.

The first step of lung nodule detection is candidate nodule detection. The nodule candidates in volume should be detected before nodule segmentation. The lung volume includes vessels and nodules. Moreover, the density of nodules, vessels, and lungs is different from each other [26]. Since the lung nodules have a circular and helical structure, they can be differentiated by means of circularity determination. Many methods have been suggested for identifying the round objects. Circular Hough transform (CHT), which proposed by Duda et al. [27], is one of the most successful method [28] for detection of round objects on the images. In this study, CHT operations are used for candidate nodule detection. CHT can detect the round object in the image; moreover, it can also detect the noncircular object by means of some operations. The image dataset is divided into 3 categories according to the nodule size such as <10 mm, 10–20 mm, and >20 mm. In order to detect the nodules in different size by CHT, three minimum and maximum radiuses such as 3–12 mm, 10–20 mm, and 15–45 mm are determined. In Figure 3, it is shown that the examples of determination of candidate nodules on CT images.

**Figure 4:**
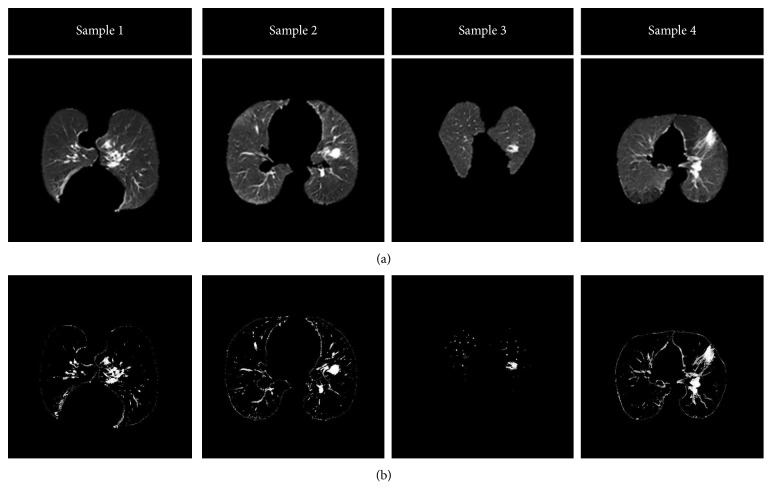
Examples of segmented lung nodules: (a) images of extracted lung volume and (b) segmentation of lung nodule using SOM.

The second step of lung nodule detection is nodule segmentation. In this study, SOM [29] is proposed to segment nodules on CT images. SOM is an unsupervised neural network learning [30] method. It can perform on large/complex datasets [31, 32]. Furthermore, it designs data maps that can be interpreted easily. In addition to these advantages, SOM can easily segment very small nodules on the lung CT images [3]. The examples of segmented lung nodule images using SOM are shown in Figure 4.

### 3.3. Nodule Feature Computation

Generally, CAD systems segment lung nodules for the determination of nodule candidates, and then features extract from the candidate nodules. The popular features are geometric feature, gray level features, gradient features, and energy level features. Therefore, we extracted 2D significative features from lung CT images to discriminate benign or malign nodule. Firstly, we used shape-based features for analyzing nodule geometry. We used first-order statistical features to obtain global statistic about nodule region. Moreover, we utilized gray level co-occurrence matrix (GLCM) texture features for gray level statistic of nodules. Finally, we extracted wavelet decomposition transform features to obtain the energy feature of nodules. All computed features are extracted from the slice of the segmented object.

First-order statistical features (SSF) of an image are calculated from the gray level histogram values of an image [33]. In this study, 6 basic features such as standard deviation, entropy, means, skewness, kurtosis, and variance were extracted by SSF using the histogram values of a gray level lung CT image. Shape-based features (SBF) allow feature extraction from the image by using geometric parameters [34]. Shape features give some information about an image such as sharpness, circularity, and convexity. In this study, a total of 16 shape features were extracted to facilitate the determination of nodule type from CT lung images. Statistical features of a gray level image (GTF) of a texture are first derived with the help of GLCM texture features proposed by Haralick [35, 36]. GLCM method shows the relationship between pixels of different gray level and is widely used in applications of medical image processing. In this study, a total of 88 features were extracted with GLCM from 0°, 45°, 90°, and 135° angle directions in *d* = 2 distance. Wavelet decomposition transform can denote distribution of energy features of different regions (TEF). ROI of the CT image is divided into four subbands with 2D wavelet decomposition. Three images are created in low frequencies, and an image is created in high frequencies with wavelet decomposition transform from an image [37]. In this study, 13 energy features of an image are extracted with wavelet decomposition. The number of features extracted by each feature extraction method used in this study is presented in Table 1.

**Figure 5:**
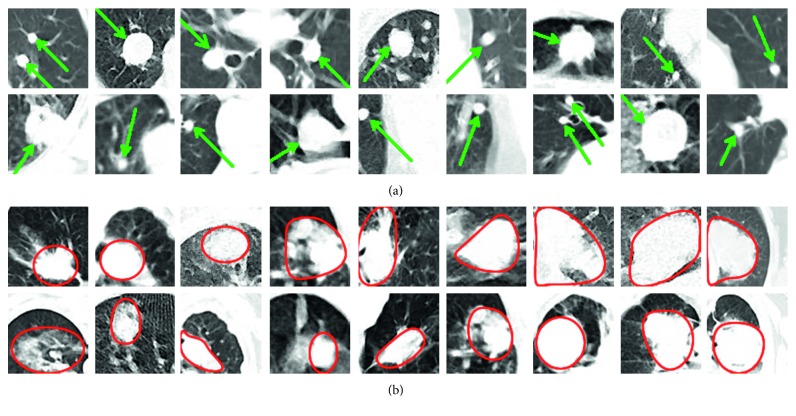
Examples of benign (a) and malign (b) lung nodules.

On lung CT images, malign nodules are generally more complex and irregular, while benign nodules are rounder with certain borders. Most of the benign nodules have small variance values. However, malign nodules show relatively higher variance values [3].  Figure 5 shows the examples of benign and malign lung nodules on CT images.

**Figure 6:**
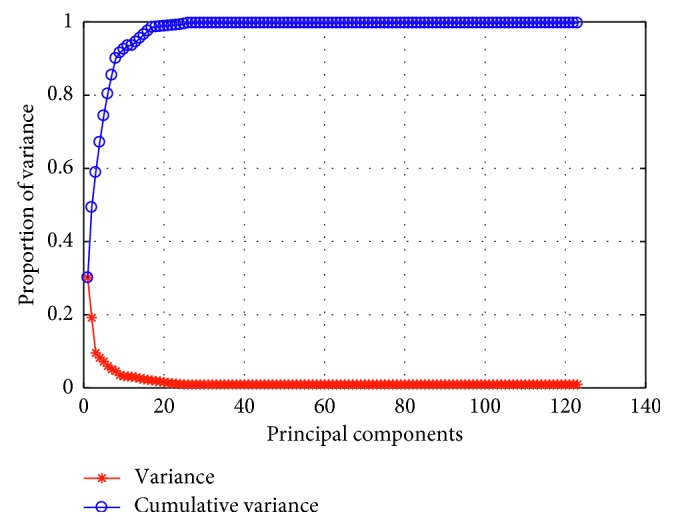
Principal component analysis of extracted features with cumulative variance.

Since 123 features extracted are rather large in size, they may negatively affect accuracy during classification step. Thus, selecting the most appropriate features instead of using all features will be a more efficient method. We used PCA method for dimension reduction of feature vector. PCA is used to reduce dimensionality of large dataset [38, 39]. We can select a number of features only up to one-third of the number of data (patterns) in the smaller of the two classes. Thus, for our work, the smallest class has 104 patterns (benign nodules), and since we split the data to half, one-third of 52 is around 17. Therefore, we selected with PCA the most appropriate 17 features (components) from 123 features.  Figure 6 denotes principal component analysis of extracted features with cumulative variance. As can be seen from the chart in Figure 6, it is seen that the variance of the first 20 components is more selective.

### 3.4. Nodule Classification

**Figure 7:**
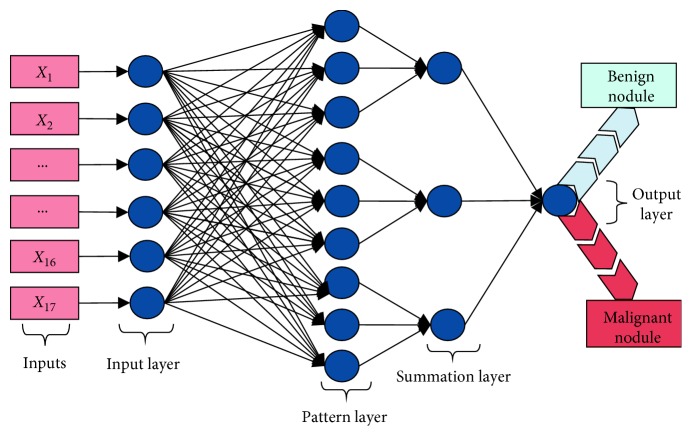
Probabilistic neural network architecture used in the proposed method for nodule classification.

In the proposed pipeline, we have used a probabilistic neural network (PNN) model to make automated decision about the nodule types (benign or malignant). PNN is an effective tool for many classification implementations and can easily make classifications [40, 41].  Figure 7 presents the architecture of the PNN designed for this study. Neuron number in the input layer is selected 17 according to the number of inputs.

## 4. Experimental Results

**Figure 8:**
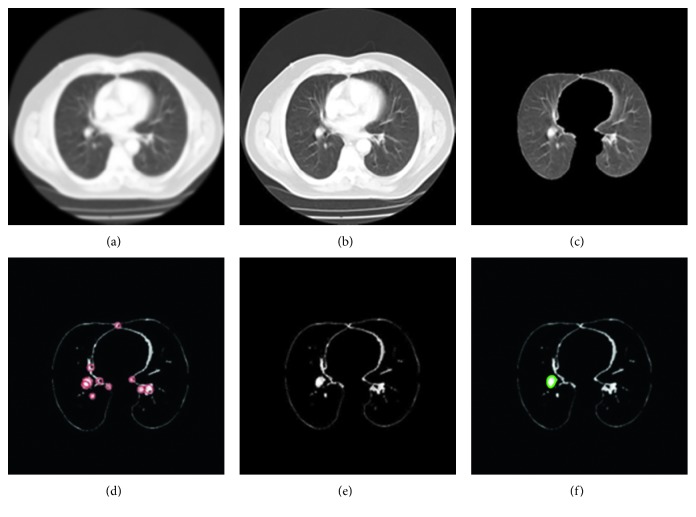
Processing steps of the proposed pipeline: (a) original DICOM image; (b) image preprocessing and enhancement; (c) lung volume extraction from CT scan; (d) detection of candidate nodules; (e) segmentation of nodules; (f) classification of nodules.

In this work, we realized all experiments using a PC with i7 processor, 16 GB memory, and Windows 10. Moreover, MATLAB software was used for performance evaluation of the proposed pipeline. In all experiments, leave-one-out cross validation technique was run at the level of nodule. So, all of 220 nodules (104 benign and 116 malignant) were used for both trainings and tests.  Figure 8 summarizes the processing steps of the proposed pipeline.

### 4.1. CT Lung Dataset

**Figure 9:**
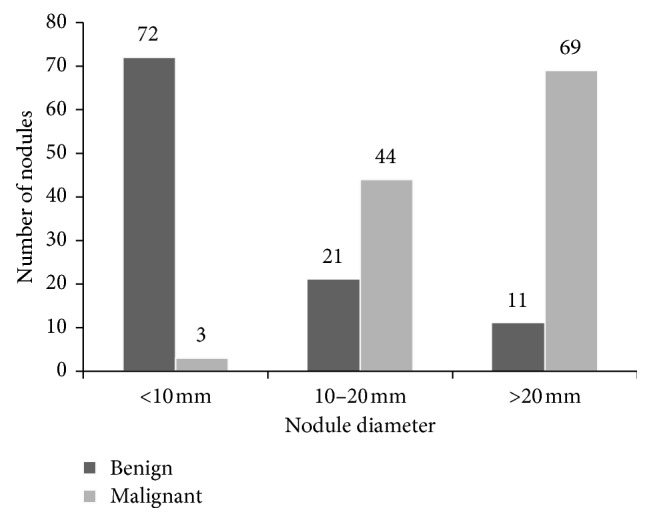
Size distribution of benign and malignant nodules in the image dataset.

In this study, an image dataset was prepared for the proposed pipeline. CT examinations were realized by using a helical CT scanner from Sincan Nafiz Korez Hospital. Its acquisition parameters are slice collimation 1.0 mm and slice width 1 mm. Scans were acquired in 130 kV and 75 mAs. The size of the images was 512 × 512 pixels. The images were stored as DICOM format files. The database consists of 47 CT scans from 47 different patients. 35 of volunteer patients are male and remaining of them are female. Their ages are between 30 and 79 (mean 58.7 ± 10.5 years). All patients agreed that they have a legal and moral right to accurate and reliable information for the scan. These patients should be given clearly the diagnosis and prognosis with a simple language. There are a total of 9504 CT modality images in the database, and the number of CT slices per scan varies between 116 and 283. After the CT scan, the physician provided the selection of the slice where the nodule is fully visible. 1128 ROI, which includes a total of 220 nodules (104 benign and 116 malignant), were selected from 9504 CT images with the help of a lung physician and three experienced radiologists in the lung parenchyma. This process has been conducted by means of an annotation tool. The nodules were also approved by biopsy. Sizes of nodules change from 3 to 65 mm in diameter. The size distribution of the nodules is shown in Figure 9.

### 4.2. Validation of LUVEM with Evaluation Metrics

Proposed lung volume extraction method (LUVEM) in this study is compared with the standard manual segmentations using measurement metrics. We evaluate manual segmentations of the expert and automated segmentations of LUVEM using two popular overlap measures. We used a segmentation software tool developed by us for manual segmentation on the dataset. The software tool outlines edges automatically, presenting us to obtain contours of the nodule boundaries. The metrics evaluate the overlapping between the two sets. The first overlap metric, represents the Jaccard coefficient (union overlap), defined as intersection over manual and automatic segmentations and measures the similarity of the S1 and S2 sets [42]. Our second overlap metric, the Dice coefficient (mean overlap), gives double the weight to agreements between the two sets [43]. Jaccard and Dice metrics are denoted in the following equations:

(1) $$\begin{array}{c} \begin{array}{c} \text{Jaccard}=\frac{\left|S1\;\cap \;S2\right|}{\left|S1\;\cup \;S2\right|},\\ \text{Dice}=\frac{2\left|S1\;\cap \;S2\right|}{\left|S1\right|+\left|S2\right|}. \end{array} \end{array}$$

We show the overlap metrics (Jaccard and Dice) that result from both LUVEM and Otsu's method. These results are the comparison of automated segmentations of LUVEM and Otsu methods with manual segmentation on 254 lung CT image in our database. The results in Table 2 showed LUVEM is higher in Jaccard overlap (0.867) and Dice overlap (0.938) than Otsu's method.

### 4.3. Detection Rates

Confusion matrixes of classification results with PNN according to each feature extraction and PCA method are presented in Table 3. As shown in Table 3, the usage of PCA affects the detection performance of the pipeline positively. Moreover, the usage of combined features extraction methods with PCA gives best success rate.

Table 4 presents the values of performance criteria obtained in the classification results of the proposed pipeline when feature extraction methods were used separately and together. According to the table, performance values are more successful when all feature extraction methods are used together. Accuracy (Acc) was found to be 92.27% when 123 features were used in classification without feature selection through PCA, and this rate was found to be 95.91% with the use of PCA. Similarly, more successful results were obtained in sensitivity (Sen), specificity (Spc), positive decision value (PDV), negative decision value (NDV), and F1 score criteria as presented in Table 4.

Since our CT image database was divided into 3 groups according to the size of nodules such as <10, 10–20, and> 20 mm, we also realized a performance evaluation according to size group of the nodules. These experiments were realized with all together feature extraction method using PCA.  Table 5 presents the result of detection performance depending upon nodule size. As shown in Table 5, the proposed pipeline can classify even small nodules with high success rates. Overall detection result of proposed pipeline according to the nodule size is 95.91%.

**Figure 10:**
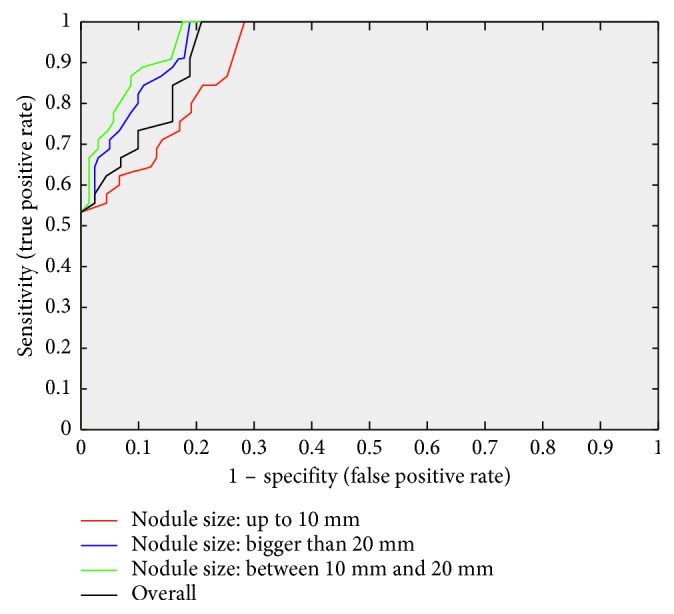
ROC curve of classification precision in proposed pipeline in different nodule diameter.

Receiver operator characteristic (ROC) curve is another popular performance evaluation criteria used in detection systems. Area under an ROC curve is measured according to sensitivity and specificity values of system. This area shows how the system is successful. Therefore, we also present ROC curve of our proposed detection system.  Figure 10 shows ROC curve of the system obtained classification results of each lung nodule group and overall system. As seen in this graphic, area under ROC curve and true positive rate of small size nodules are lower than big size nodules. Here, as can be seen from this figure, if the nodule size is too large and too small, the success rate decreases.

Processing time is another performance criterion that we have used for the evaluation of the proposed pipeline. Longest time is needed for nodule detection step due to the use of SOM method for segmentation. Since SOM is an ANN model, it has a lot of time-consuming mathematical operations. In average, classification of a CT image as benign or malignant takes 2–3 seconds approximately. It can be accepted as a reasonable time period when it is compared with the time it needs for a physician to make decisions.

## 5. Conclusions and Discussion

In this study, a fully automated pipeline was proposed to classify benign and malign lung nodules on CT images. By means of the designed pipeline, nodule detection as well as benign/malign distinction was performed with high accuracy, sensitivity and specificity rates. Moreover, it was designed a preprocessing method called LUVEM for extracting the lung volume from CT images. SOM method was used to allow successful detection of lung nodules in early stages. According to the detailed experiment performed on large dataset with combined features, the proposed pipeline can differentiate benign/malign nodules with high accuracy rates such as 94.68% (3–10 mm), 96.92% (10–20 mm), and 96.25% (>20 mm) using PNN. The proposed pipeline can be used by the physicians as a supplementary tool for benign and malign nodule classification.

We evaluated the performance of the pipeline on Lung Imaging Database Consortium-Image Database Resource Initiative (LIDC-IDRI) as well [50]. LIDC-IDRI dataset is the largest publicly available reference database for detection of lung nodules. We choose LIDC-IDRI dataset since it contains almost all the related information for lung CT including annotations on nodule sizes, locations, diagnosis results, and other information. We collected a total of 38 lung nodules from the dataset, including 26 malignant and 12 benign nodules. According to the evolution results on the proposed pipeline, accuracy obtained 84.21% using all FE methods and PCA. In this test, F1 score result was found as 0.88. The obtained performance evaluation values of proposed pipeline on LIDC-IDRI dataset are denoted in Table 6.

There are some advantages of the proposed pipeline compared to the state-of-the-art systems. Firstly, the proposed pipeline has two diagnosis possibilities. It can perform nodule detection together with nodule classification. Second advantage of the proposed pipeline is to provide the detection of small nodules in the lung with the use of SOM method during segmentation step. This is remarkable in terms of early detection of lung cancer. Third advantage of the proposed pipeline is to have relatively high detection performance. Accuracy, sensitivity, and specificity of the system were calculated as 95.91%, 97.42%, and 94.24%, respectively. It is fairly difficult to compare formerly reported CAD systems due to different datasets, nodule types, sizes, and validation methods. We picked out some CAD systems to compare their performances. Some of them [23, 24, 44–46] used the LIDC database [47–49], and the other used their own databases.  Table 7 denotes the comparison of the proposed pipeline with some CAD systems. When the results are analyzed, our pipeline has high sensitivity on our CT image dataset.

## Figures and Tables

**Algorithm 1:**
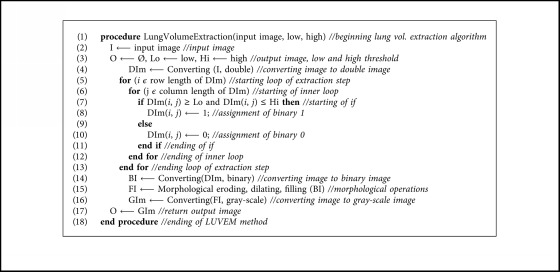
The pseudocode of lung volume extraction method (LUVEM).

**Table 1:** The number of extracted 2D features from lung CT images.

Feature extraction method	Number of feature	Order
SSF	6	0–6
SBF	16	7–22
GTF	22 ∗ 4 = 88	23–110
TEF	13	111–123

**Table 2:** The Jaccard and Dice metrics measures for LUVEM.

	Jaccard overlap	Dice overlap
Otsu's method	0.587 ± 0.093	0.786 ± 0.088
*LUVEM*	**0.867** ± **0.051**	**0.938** ± **0.032**

**Table 3:** Confusion matrixes for feature extraction methods.

FE method	Classification results without PCA				Classification results with PCA			
	TP	FP	FN	TN	TP	FP	FN	TN
SSF	90	22	26	82	90	22	26	82
SBF	105	17	11	87	109	12	7	92
GTF	101	19	15	85	110	11	6	93
TEF	109	15	7	89	111	11	5	93
All FE methods (combined)	111	12	5	92	113	6	3	98

**Table 4:** Overall performance results of proposed pipeline.

Performance criteria	Classification results without PCA					Classification results with PCA				
	SSF	SBF	GTF	TEF	All	SSF	SBF	GTF	TEF	All
Acc	78.18	87.27	84.55	90.00	92.27	78.18	91.36	92.27	90.00	95.91
Sen	77.57	90.52	87.07	93.97	95.67	77.57	93.97	94.83	95.67	97.42
Spc	78.85	83.65	81.73	85.58	88.46	78.85	88.46	89.42	89.42	94.24
PDV	80.36	86.07	84.17	87.90	90.24	80.36	90.08	90.91	90.98	94.96
NDV	79.93	88.78	85.00	92.71	94.85	79.93	92.93	93.94	94.90	97.03
F1	0.79	0.88	0.85	0.92	0.93	0.79	0.92	0.93	0.94	0.96

**Table 5:** Assessment of performance measurement criteria according to nodule size.

Nodule size (mm)	The number of nodule	Confusion matrix				Performance criteria					
		TP	FP	FN	TN	Acc	Sen	Spc	PDV	NDV	F1
<10	75	3	4	0	68	94.67	100	94.45	42.86	100	0.60
10–20	65	43	1	1	20	96.92	97.73	95.24	97.73	95.24	0.98
>20	80	67	1	2	10	96.25	97.10	90.91	98.53	83.34	0.98
Overall	220	113	6	3	98	95.91	97.42	94.24	94.96	97.03	0.96

**Table 6:** The performance evaluation of proposed pipeline on LIDC-IDRI.

The number of nodules	Confusion matrix				Performance criteria					
	TP	FP	FN	TN	Acc	Sen	Spc	PDV	NDV	F1
38	22	2	4	10	84.21	84.62	83.33	91.67	71.43	0.88

**Table 7:** The comparison of our pipeline with previously published CADs.

CAD system	CT image database	Number of cases	Nodule size(mm)	Sensitivity (%)	Average FPR
Dehmenski et al. [9]	Their own database	70	3–20	90.0	14.6
Suarez-Cuenca et al. [10]	Their own database	22	4–27	80.0	7.7
Opfer and Wiemeker [46]	LIDC database [47, 48, 50]	93	≥4	74.0	4
Rubin et al. [51]	Their own database	20	≥3	76	3
Sahiner et al. [49]	LIDC database [47, 48, 50]	48	3–36.4	79	4.9
Messay et al. [24]	LIDC database [47, 48, 50]	84	3–30	82.66	3
Suzuki et al. [52]	Their own database	101	8–20	80.3	16.1
Park et al. [53]	Their own database	38	Indefinite	80	–
Choi and Choi [23]	LIDC database [47, 48, 50]	32	3–30	94.1	5.45
Choi and Choi [44]	LIDC database [47, 48, 50]	58	3–30	95.28	2.27
Proposed method	Our database	47	3–35	97.42	4.54

## Data Availability

The data used to support the findings of this study are available from the corresponding author upon request.
